# The efficacy and safety of lebrikizumab monotherapy for the management of moderate-to-severe atopic dermatitis: A systematic review and meta-analysis

**DOI:** 10.3389/fmed.2022.1091271

**Published:** 2023-01-16

**Authors:** Bader Bashrahil, Ziyad Alzahrani, Sahal Samarkandy, Abdullah Aman, Abdulhadi Jfri

**Affiliations:** ^1^College of Medicine, King Saud bin Abdulaziz University for Health Sciences, Jeddah, Saudi Arabia; ^2^King Abdullah International Medical Research Center, Jeddah, Saudi Arabia; ^3^Department of Dermatology, Ministry of the National Guard-Health Affairs, Jeddah, Saudi Arabia

**Keywords:** atopic dermatitis, eczema, lebrikizumab, anti-IL-13, monoclonal antibodies

## Abstract

**Background:**

Atopic dermatitis (AD) is a chronically relapsing disease. Few biologics are approved for moderate-to-severe AD, and novel interventions are emerging. We aimed to evaluate the safety and efficacy of lebrikizumab, an IL-13 immunomodulator, as monotherapy vs. placebo in treating moderate-to-severe AD.

**Methods:**

Cochrane Central Register of Controlled Trials (CENTRAL), Medline, Embase, and ClinicalTrials.gov registry (CT.gov) databases were systematically searched. We evaluated lebrikizumab vs. placebo and measured efficacy using Eczema Area and Severity Index (EASI), Body Surface Area (BSA), and Investigator’s Global Assessment (IGA) change from baseline to week 16. Safety was evaluated by the incidence of serious adverse events (SAEs), non-serious adverse events (NSAEs), and mortality. The risk of bias was investigated using the Revised Cochrane risk of bias tool.

**Results:**

Three RCTs (*n* = 1,149) included 543 (47.25%) men vs. 606 (52.75%) women. Meta-analysis showed statistically significant improvement in EASI, IGA, and BSA. EASI75 at week 16 for all regimens was (RR = 2.62, 95% CI [2.06, 3.34], *p* < 0.00001) with the first regimen (500 mg loading dose then 200 mg every 2 weeks) showing the most significant improvement (RR = 3.02, 95% CI [2.39, 3.82], *p* < 0.00001). The pooled analysis of safety outcomes concluded that lebrikizumab did not correlate significantly with the incidence of SAEs, NSAEs, and mortality.

**Conclusion:**

Overall, lebrikizumab showed a significant improvement in all efficacy outcomes. Additionally, it did not contribute to any significant incidence of SAEs, NSAEs, or mortality. The risk of bias in included RCTs was minor except in the randomization domain. Grading of Recommendations Assessment, Development, and Evaluation (GRADE) assessment of the outcomes ranged from low to high, but predominantly high certainty of evidence.

**Systematic review registration:**

https://www.crd.york.ac.uk/prospero/, identifier CRD42022362438.

## Introduction

Atopic dermatitis (AD) is a chronic inflammatory pruritic skin disease with a worldwide prevalence estimated to be as high as 20% (1). It mainly affects the face, neck, arms, and legs, but less commonly affects the groin and the axilla (2). AD is predominantly diagnosed in childhood; however, the disease can occur or relapse later in life (3). Topical treatments are the mainstay of treatment along with general skin care measures, but they have shown multiple limitations and minimal therapeutic effects, especially in more severe forms of AD (4). Multiple theories have attempted to elucidate AD’s pathophysiology. One of these had associated AD with elevated serum immunoglobulin E (IgE) levels and T-helper cells type 2 (Th2) mediated inflammation that predisposes to interleukin-4 (IL-4) and interleukin (IL-13)-driven pathology. Novel drugs are emerging to target these cytokines as management options for AD (5). Janus kinase (JAK) inhibitors and monoclonal immunomodulators such as abrocitinib, a JAK1 inhibitor, and dupilumab, an IL-4 inhibitor, were recently established as effective treatments and have received the US Food and Drug Administration (FDA) approval for managing moderate-to-severe AD. JAK inhibitors are mostly for oral use and one of which, tofacitinib, is additionally used as a topical off-label option (6). Dupilumab was approved by the FDA in 2017 and set the stage for more IL immunomodulators to emerge (6, 7). Tralokinumab and lebrikizumab are the latest drugs attempting to target IL-13 to minimize AD signs and symptoms (8). Despite both drugs having been investigated in recent studies, more evidence is needed to establish them as a main line of treatment. Tralokinumab has been investigated in multiple studies since it was the initial drug in its class; however, lebrikizumab monotherapy is nearly an intact subject, especially in studies with high levels of evidence (9). In this article, the comprehensive systematic review and meta-analysis of randomized controlled trials (RCTs) were performed to centrally assess the safety and efficacy of lebrikizumab for managing moderate-to-severe AD against a placebo.

## Methods

Our study was registered prior to a preliminary search in alignment with PROSPERO (CRD42022362438). It utilized the Preferred Reporting Items for Systematic Reviews and Meta-Analysis (PRISMA) checklist.

### Eligibility criteria

Our systematic review and meta-analysis exclusively included RCTs that compared lebrikizumab against a placebo in patients with moderate-to-severe AD who are older than 12 years. Studies that did not match any of the aforementioned outcome variables were excluded. RCTs that had their patients undergo calcineurin inhibitors, topical corticosteroids, or phosphodiesterase-4 inhibitors 1 week prior to baseline or that used any systemic treatments such as JAK inhibitors, systemic corticosteroids, or phototherapy 4 weeks before baseline assessment were not included.

### Search strategy

We systematically searched the Medline, Embase, ClinicalTrials.gov (CT.gov), and Cochrane Central Register of Controlled Trials (CENTRAL) databases from database initiation to 1 October 2022 without any restriction on date or language. The search strategy is provided in the Supplementary appendix. References of the included RCTs were inspected for relevant RCTs that were missed during the systematic search process.

### Study selection and data extraction

Independently, two reviewers (BB and ZA) performed title and abstract screening, full-text assessment, and data extraction of RCTs that match the eligibility criteria. Disputes of studies’ inclusion or exclusion were resolved with a third senior author opinion (AJ).

### Outcomes

Efficacy outcomes of this article included percentage (%) change from baseline in Eczema Area and Severity Index (EASI), Body Surface Area (BSA), and the number of participants with an Investigator’s Global Assessment (IGA) score of 0 (clear) or 1 (almost clear) and a reduction of ≥2 points or a reduction in EASI scores that is more or equals 75% (EASI75). In the included studies, IGA and EASI75 reported only percentages of patients fulfilling the outcome. Thus, the percentage of participants achieving these two outcomes was converted into a dichotomous number of participants using their percentages and the corresponding number of analyzed patients. Decimal numbers of participants had shown during the calculation, moreover, and were rounded to the most proximal whole number and had their *p*-value checked for matching with the studies’ results. Week 16 is the time point of evaluation for the efficacy outcomes. The safety profile of lebrikizumab was evaluated by measuring the incidence of serious adverse events (SAEs), non-serious adverse events (NSAEs), and mortality.

### Meta-analysis

Data analysis was performed using RevMan (Review Manager) version 5.3 (Cochrane Collaboration). All statistical analyses were performed using the random-effects model. A 95% confidence level and *p* < 0.05 as a borderline were set for statistical significance. The statistical heterogeneity was assessed using the I^2^. Percent change in EASI and BSA at week 16 were the only continuous variables, and the standardized mean difference (SMD) was used to measure their effects. Dispersion of data was measured using standard deviation (SD). RCTs that used standard error (SE) instead of SD had SE converted to SD by multiplying SE in the squared root of the corresponding sample size. Dichotomous outcomes (SAEs, NSAEs, and mortality) were demonstrated as risk ratios (RRs) and pooling was performed using the inverse variance (IV) weighting method. Data were classified into three subgroups in the subgroup analysis to measure the effects of different regimens. The first regimen included patients that were given 500 mg as a loading dose at baseline and 250 mg every 2 weeks (Q2W). The second regimen included patients that were injected with 250 mg at baseline and 125 mg of lebrikizumab every 4 weeks (Q4W). The third regimen included patients that were administered a 500 mg loading dose and 250 mg of lebrikizumab Q4W. The quality of evidence of outcomes was assessed using the Grading of Recommendations Assessment, Development, and Evaluation (GRADE) criteria.

## Results

**FIGURE 1:**
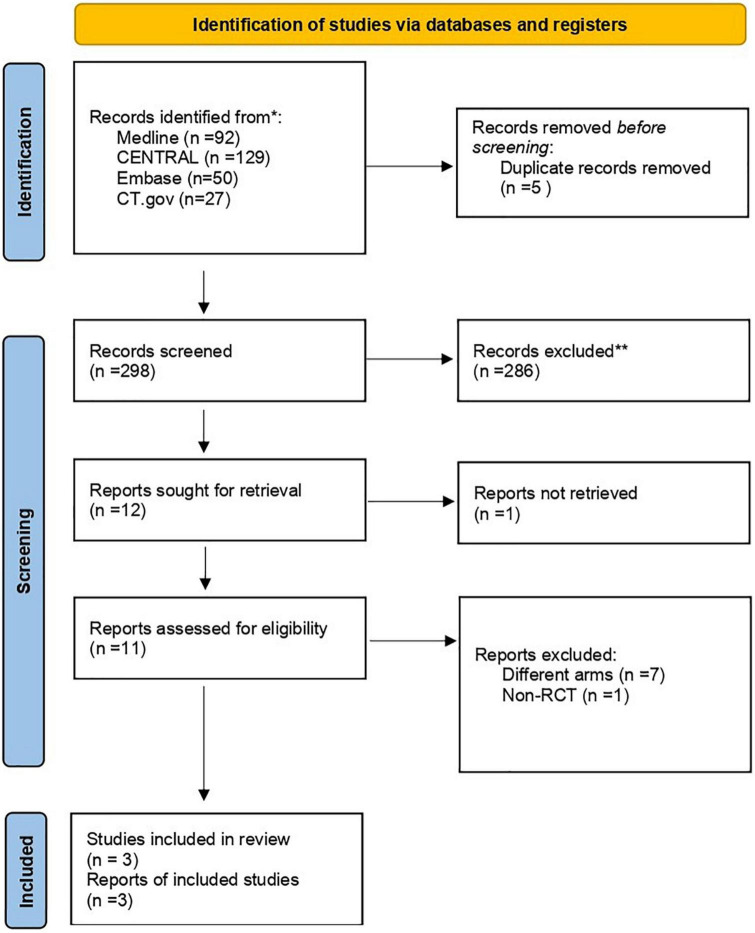
Study flowchart as per the Preferred Reporting Items for Systematic Reviews and Meta-Analysis (PRISMA) criteria. CENTRAL, Cochrane Central Register of Controlled Trials; RCT, randomized controlled trial. *Search results on October 1, 2022. **Exclusion was done by humans exclusively.

After a systematic search, 298 studies were found, with 5 duplicates initially removed. Screening resulted in 12 studies that were assessed for eligibility (Figure 1). Of the 12 studies, one was unobtainable while eight were excluded due to unmatched eligibility. Eventually, three studies remained that represented RCTs that were included in our study. All populations, interventions, comparisons, and outcomes of these RCTs matched our eligibility criteria.

### Trial characteristics

Included studies (*n* = 3) assessed 1,149 participants of both arms (Table 1). The lebrikizumab arm comprised 806 participants, while the placebo arm contained 343 participants. Two studies had patient age as groups and recorded 105 patients that were 18 years or younger, 700 patients between 18 and 65 years, and 64 patients that were 65 years or older. One study had age as mean ± SD (39.3 ± 17.48) (10–12). Of the 1,149 participants, 543 were men and 606 were women.

**TABLE 1:** Characteristics of included trials.

CT.gov identifier	Lebrikizumab regimen	Number of participants (started)		Number of participants (completed)		Age group			Gender	
		Lebrikizumab	Placebo	Lebrikizumab	Placebo	≤18 years	Between 18 and 65 years	≥65 years	Male	Female
NCT04178967	500 mg lebrikizumab (2 × 250 mg) SC injections as a loading dose at baseline and week 2 visits followed by a single 250 mg lebrikizumab injection Q2W from week 4 until week 14.	295	150	273	133	50	362	33	219	226
NCT04146363	500 mg lebrikizumab (2 × 250 mg) SC injections as a loading dose at baseline and week 2 visits followed by a single 250 mg lebrikizumab injection Q2W from week 4 until week 14	283	141	263	120	55	338	31	210	214
NCT03443024	250 mg lebrikizumab (2 × 125 mg) SC injections as a loading dose at baseline followed by a single 125 mg lebrikizumab Q4W from week 4 until week 14	73	52	52	20	39.3 ± 17.48			114	166
	500 mg lebrikizumab (4 × 125 mg) SC injections as a loading dose at Baseline followed by a single 125 mg lebrikizumab Q4W from week 4 until week 14	80	52	49	20					
	500 mg lebrikizumab (2 × 250 mg) SC injections as a loading dose at baseline and week 2 visits followed by a single 250 mg lebrikizumab injection Q2W from week 4 until week 14.	75	52	56	20					

CT.gov, clinicaltrials.gov registry; mg, milligram; SC, subcutaneous; Q2W, every 2 weeks; Q4W, every 4 weeks.

### Risk of bias assessment

**FIGURE 2:**
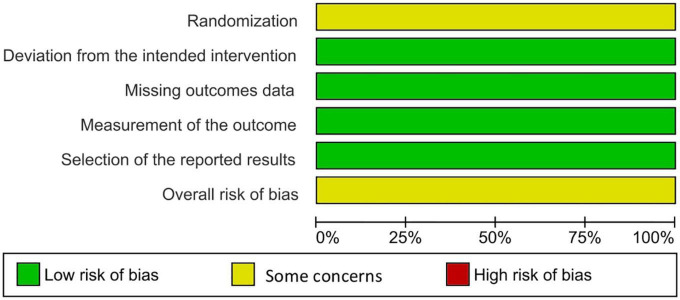
Risk of bias graph.

**FIGURE 3:**
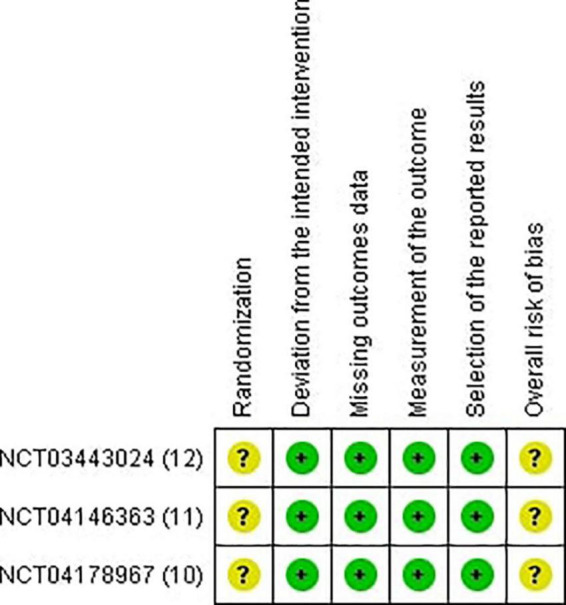
Risk of bias summary.

Two reviewers independently utilized the Revised Cochrane risk of bias tool to assess the risk of biases in the evaluated RCTs. Individual studies were reviewed and rated as high risk, low risk, or some concerns. Disagreements between the reviewers were resolved through discussion until a final decision was reached (13) (Figures 2, 3).

### Efficacy outcomes

#### The percentage change in EASI score

**FIGURE 4:**
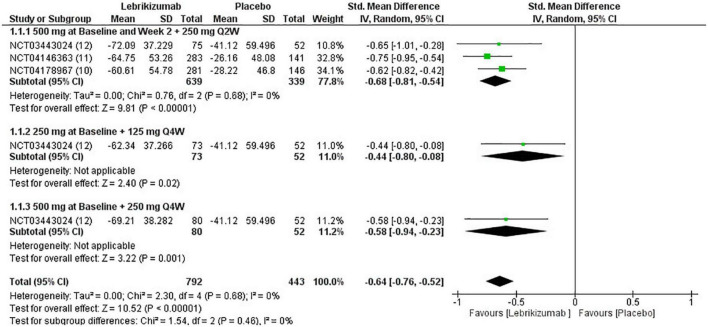
Forest plot of EASI score. CI, confidence interval; IV, inverse variance; SMD, standardized mean difference; SD, standard deviation; Q2W, every 2 weeks; Q4W, every 4 weeks.

**FIGURE 5:**
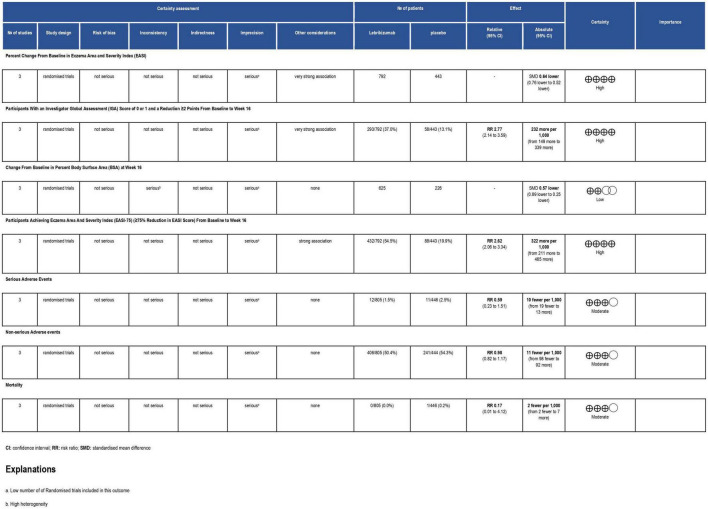
Grading of Recommendations Assessment, Development and Evaluation (GRADE) evidence profile. CI, confidence interval; RCT, randomized controlled trial; RR, risk ratio.

The three included studies measured percent change in EASI score from baseline at different time points. Our study exclusively evaluated the percent change from baseline at their visit on week 16. All three regimens had a significant reduction in EASI score (SMD = −0.64, 95% CI [−0.76, −0.52], *p* < 0.00001, I^2^ = 0%). The first regimen had the most superior effect, followed by the third and the second subgroups, respectively (SMD = −0.68, 95% CI [−0.81, −0.54], *p* < 0.00001, I^2^ = 0%) (SMD = −0.44, 95% CI [−0.80, −0.08], *p* = 0.02, I^2^ = not applicable) (SMD = −0.58, 95% CI [−0.94, −0.23], *p* = 0.001, I^2^ = not applicable) (Figure 4). Certainty of evidence of GRADE criteria was estimated to be high (Figure 5).

#### IGA score

**FIGURE 6:**
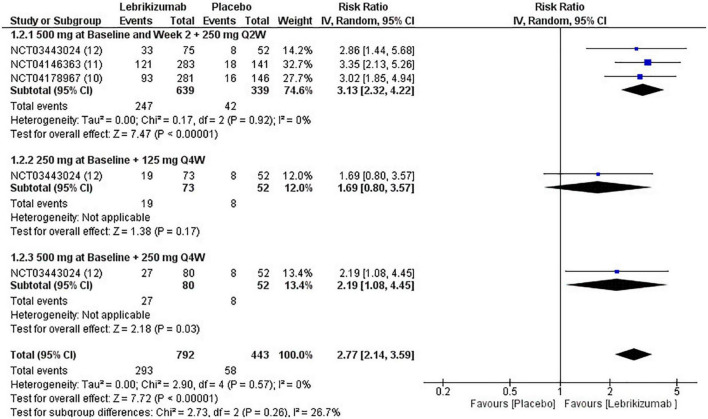
Forest plot of IGA score. CI, confidence interval; IV, inverse variance; RR, risk ratio; Q2W, every 2 weeks; Q4W, every 4 weeks.

All three RCTs evaluated IGA at week 16. The first subgroup (RR = 3.13, 95% CI [2.32, 4.22], *p* < 0.00001, I^2^ = 0%) and the third regimen (RR = 2.19, 95% CI [1.08, 4.45], *p* = 0.03, I^2^ = not applicable) showed a significant number of participants achieving score 0 and 1 or a reduction of 2 or more points from baseline to 16 weeks. However, the second subgroup did not improve this outcome significantly (RR = 1.69, 95% CI [0.80, 3.57], *p* = 0.17, I^2^ = not applicable). Overall, lebrikizumab had significant improvement in IGA outcome (RR = 2.77, 95% CI [2.14, 3.59], *p* < 0.00001, I^2^ = 0%) (Figure 6). GRADE assessment of this outcome was found to be high (Figure 5).

#### BSA score

**FIGURE 7:**
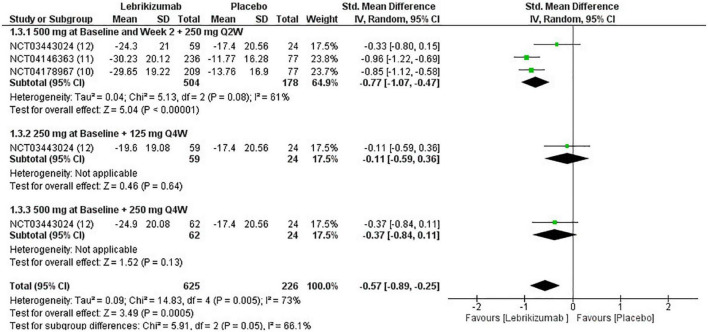
Forest plot of the percentage change in BSA. CI, confidence interval; IV, inverse variance; SMD, standardized mean difference; SD, standard deviation; Q2W, every 2 weeks; Q4W, every 4 weeks.

Included RCTs (*n* = 3) had a significant deduction of BSA involved with AD (SMD = −0.57, 95% CI [−0.89, −0.25], *p* = 0.0005, I^2^ = 73%). However, this improvement pertained to the first regimen (SMD = −0.77, 95% CI [−1.07, −0.47], *p* < 0.00001, I^2^ = 61%) since the second and the third subgroups did not correlate with a remarkable reduction in this efficacy outcome (SMD = −0.11, 95% CI [−0.59, 0.36], *p* = 0.64, I^2^ = not applicable) (SMD = −0.37, 95% CI [−0.84, 0.11], *p* = 0.13, I^2^ = not applicable) (Figure 7). BSA score had a low certainty of evidence in GRADE evaluation (Figure 5).

#### EASI75

**FIGURE 8:**
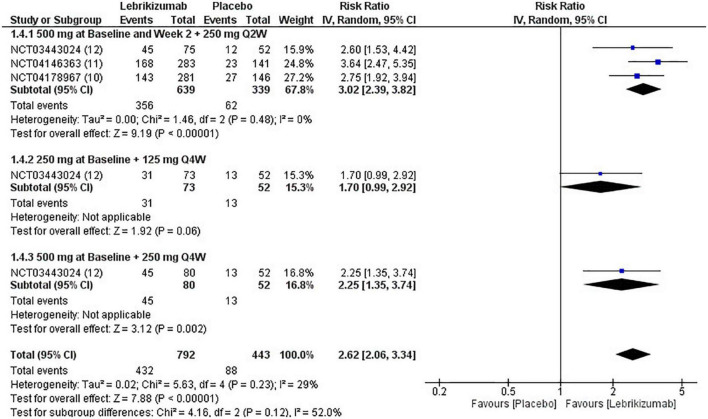
Forest plot of EASI75 score. CI, confidence interval; IV, inverse variance; RR, risk ratio; Q2W, every 2 weeks; Q4W, every 4 weeks.

The three included RCTs had a significantly pooled number of participants achieving more than or equal to a 75% reduction in EASI score from baseline to week 16 (RR = 2.62, 95% CI [2.06, 3.34], *p* < 0.00001, I^2^ = 29%). The first regimen had the most significant improvement (RR = 3.02, 95% CI [2.39, 3.82], *p* < 0.00001, I^2^ = 0%) while the third subgroup had a less significant effect (RR = 2.25, 95% CI [1.35, 3.74], *p* = 0.002, I^2^ = not applicable). The second subgroup did not have a sufficient number of participants to have an outcome consistent with the other regimens (RR = 1.70, 95% CI [0.99, 2.92], *p* = 0.006, I^2^ = not applicable) (Figure 8). GRADE criteria scored high certainty of evidence (Figure 5).

### Safety outcomes

#### Serious adverse events

**FIGURE 9:**
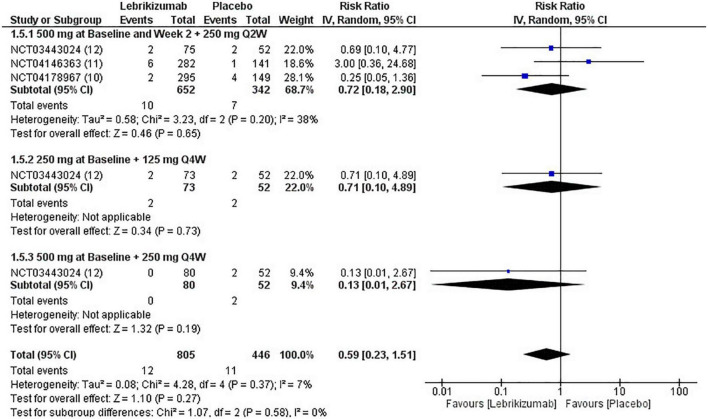
Forest plot of SAEs. CI, confidence interval; IV, inverse variance; RR, risk ratio; Q2W, every 2 weeks; Q4W, every 4 weeks.

Lebrikizumab did not cause a significant incidence of SAEs in any of the included studies (*n* = 3). It had a comparable effect of inducing SAEs that nearly matched to placebo (RR = 0.59, 95% CI [0.23, 1.51], *p* = 0.27, I^2^ = 7%). Both the first (RR = 0.72, 95% CI [0.18, 2.90], *p* = 0.65, I^2^ = 38%) and the second (RR = 0.71, 95% CI [0.10, 4.89], *p* = 0.73, I^2^ = not applicable) had similar RR, but the effect size is more considerable due to higher number of participants. Finally, the third regimen’s findings were consistent with that of the previous subgroups (RR = 0.13, 95% CI [0.01, 2.67], *p* = 0.19 I^2^ = not applicable) (Figure 9). SAE outcome was rated as moderate in the GRADE assessment (Figure 5).

#### Non-serious adverse events

**FIGURE 10:**
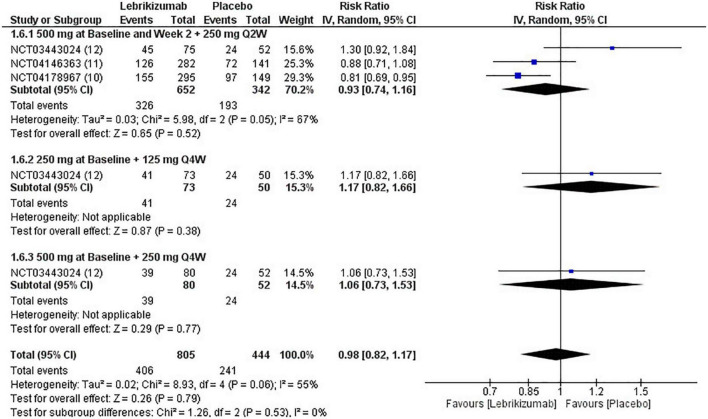
Forest plot of NSAEs. CI, confidence interval; IV, inverse variance; RR, risk ratio; Q2W, every 2 weeks; Q4W, every 4 weeks.

In all three RCTs, lebrikizumab did not record a substantial difference in its effect to induce NSAEs in comparison with placebo (RR = 0.98, 95% CI [0.82, 1.17], *p* = 0.79 I^2^ = 55%). The first regimen showed negative RR but the second and third subgroups each had a positive RR, all of which were insignificant (RR = 0.93, 95% CI [0.74, 1.16], *p* = 0.52 I^2^ = 67%), (RR = 1.17, 95% CI [0.82, 1.66], *p* = 0.38 I^2^ = not applicable), (RR = 1.06, 95% CI [0.73, 1.53], *p* = 0.38 I^2^ = not applicable) (Figure 10). Upon GRADE evaluation, NSAEs had moderate evidence certainty (Figure 5).

#### Mortality

**FIGURE 11:**
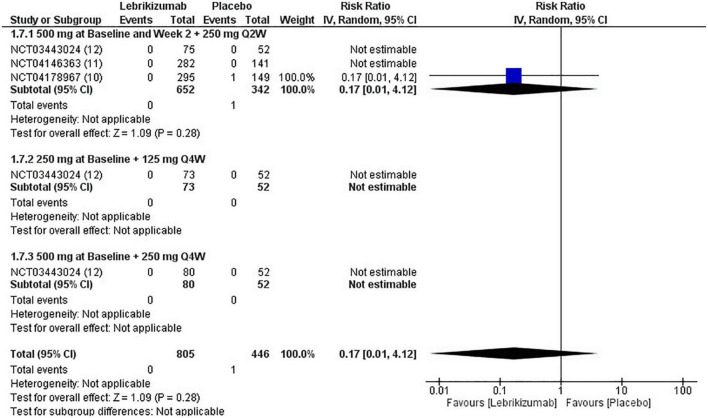
Forest plot of mortality. CI, confidence interval; IV, inverse variance; RR, risk ratio; Q2W, every 2 weeks; Q4W, every 4 weeks.

Only one study reported a single death in the placebo arm compared to the first regimen. This led to the pooled analysis being associated only with this event (RR = 0.17, 95% CI [0.01, 4.12], *p* = 0.28 I^2^ = not applicable). Neither the second nor the third arm had a reported death, which made statistical variables, by extension, not estimable (Figure 11). Mortality showed moderate certainty of evidence in GRADE criteria (Figure 5).

## Discussion

This systematic review and meta-analysis assessed the safety and efficacy of lebrikizumab for the management of moderate-to-severe AD as a monotherapy. Lebrikizumab, an IL-13 inhibitor, demonstrated an improvement in all efficacy measures predominantly in the first regimen. NSAEs and SAEs showed a comparable risk of occurrence in both the placebo and the intervention arm. Mortality was nearly non-existent in all analyzed participants.

Atopic dermatitis is mainly managed with topical agents either as monotherapy or as an adjunctive option (14). AD management options are settled to tackle multiple pathologic facets, as this disease varies in natural history, manifestations, and morphology (14, 15). Topical corticosteroids (TCSs) are highly effective options among topical treatments and have been the primary treatment for this disease for a long time (16, 17). They have a potent effect in reducing signs and symptoms of AD along with a relatively safe adverse events profile (14). This profile consisted of purpura and skin atrophy and other side effects that are mostly attributed to their steroidal nature, which also limits the ability to prescribe TCS for a timely course of treatment (18). Due to these obstacles, an alternative topical option, topical calcineurin inhibitors (TCIs), was introduced early in this millenium (19). TCIs are prescribed as a second line of treatment and are indicated in the conditions where a physician is considering or hedging against steroidal side effects (19, 20). Burning or stinging sensations are widely reported in TCIs, but they normalize later in treatment (21, 22). In addition to pharmacological options, non-pharmacological ones have mostly included bathing and moisturizing, which improved the disease’s course to a limited degree (23, 24). In more severe forms of AD, phototherapy can be used. Additionally, cyclosporine, methotrexate, azathioprine, and mycophenolate mofetil are immunotherapies that have been used, but they are less favorable choices due to broad targets and side effects. All those mentioned above necessitated AD-specific therapy (25). Biologics in AD are usually identified as JAK inhibitors including upadacitinib, abrocitinib, and baricitinib or monoclonal antibody immunomodulators targeting interleukins (ILs). Biologics are mainly administered as subcutaneous, oral, and less commonly, topical routes. The recent FDA approval of abrocitinib as an oral AD management option made it only the second oral intervention after the conventional prednisone (26). JAK inhibitors act by modulation of JAK/signal transducers and activators of transcription (STAT) pathway that have a key role in the regulation of the immune response attributed to AD and shown high efficacy with low adverse events (27, 28). ILs that have been targeted in an attempt to neutralize AD were IL-4 modulated by dupilumab, tralokinumab resembling IL-13 inhibitors, and lebrikizumab, the subject of our study (29). In a recent systematic review and meta-analysis, IL-13 inhibitors were compared to placebo (8). Tralokinumab showed a superior effect to lebrikizumab that was significant in participants achieving EASI75 and IGA 0 to 1. In EASI75, lebrikizumab outcomes resulted in a lower RR (RR = 1.69, 95% CI [1.11, 2.58], *p* = 0.01, I^2^ = 44%) than tralokinumab (RR = 1.79, 95% CI [1.26, 2.53], *p* = 0.001, I^2^ = 82%). These results are deemed to be mild to highly heterogeneous due to high I^2^. IGA score wise, tralokinumab (RR = 1.78, 95% CI [1.14, 2.80], *p* = 0.001, I^2^ = 0%) and lebrikizumab (RR = 1.75, 95% CI [1.40, 2.20], *p* = 0.001, I^2^ = 0%) showed very similar homogenous effects. Changes in EASI in lebrikizumab were insignificant, but (MD = −17.13, 95% CI [−34.40, 0.14], *p* = 0.05, I^2^ = 66%) tralokinumab showed the opposite result (MD = −21.40, 95% CI [−36.20, −6.61], *p* = 0.005, I^2^ = 96%); however, their effects were extremely heterogeneous and not explained by subgroup analysis. One of the RCTs that was analyzed in their systematic review and meta-analysis studied lebrikizumab’s viability vs. placebo, but the corticosteroids had been used at baseline in all participants. This may have led to confounding results, as corticosteroids are used as first-line management for moderate-to-severe AD. It was not included in our study since our criteria excluded any studies in which participants used corticosteroids (8).

Randomized controlled trials included in this review measured numerous outcomes, but several of them were appraised in relation to the author’s point of view and the outcomes’ clinical significance. Our results conclude that lebrikizumab is superior to a placebo for treating AD in all efficacy and safety outcomes assessed in this study. Subgroup analysis in this study showed an enormously important point regarding the effect of different regimens of lebrikizumab. The first regimen contributed to as much as 77.8% of effect weight in the efficacy variables and was the major source of significance. It was the only regimen to enhance AD outcomes in all efficacy variables. The second subgroup was the least effective drug and had a negligible effect that was only positive in EASI. The third subgroup scored a remarkable efficacy in most subgroups, except in the BSA outcome. SAEs are defined as any events that result in death or a life-threatening condition that necessitates inpatient hospitalization, where prolongation of an existing hospitalization causes patient incapacitation or that causes an anomaly of or birth defect in the participants’ offspring. NSAEs are adverse events that are not considered SAEs. All-cause mortality incidence was reported as well. Lebrikizumab exhibited absolute tolerability in all regimens and safety outcomes in this research. With regard to the risk of bias, the three studies were downgraded due to some concerns in the randomization sequence regarding the differences in the number of participants and randomized patients in the placebo arm had been considerably large. Funnel plots for publication bias were not generally assessed because the number of reports included was less than 10. GRADE assessment is a criterion that evaluates the certainty of evidence in different outcomes. It takes the study’s design and its numbers, imprecision, inconsistency, indirectness, and risk of bias. Clinical judgment is an inseparable part of GRADE evaluation.

Multiple limitations were encountered in our systematic review and meta-analysis, and a low number of RCTs was one. Only three RCTs were found due to the novelty of the drug. The first subgroup included three studies while the remaining yielded one report per each. Speculatively, this is due to the fact that the study assessing the three regimens found the first one to be the most efficacious. Consequently, this led the manufacturing pharmaceutical company to adapt this treatment plan in the following RCTs, which made subgroups in our meta-analysis substantially unbalanced.

## Conclusion

Ultimately, lebrikizumab was shown to be a promising option for treating moderate-to-severe AD. It indicated great efficacy in multiple outcomes and displayed a solid safety profile. Nevertheless, real-life experience studies are needed, as well as further trials to compare lebrikizumab to lebrikizumab with topical corticosteroids and to current management options including dupilumab and tralokinumab.

## Data availability statement

The datasets presented in this study can be found in online repositories. The names of the repository/repositories and accession number(s) can be found in the article/Supplementary material.

## Author contributions

BB and ZA performed the search and the statistical analysis. BB wrote the Introduction and the Methods. SS wrote the Results. AA wrote the Discussion and the Conclusion. AJ reviewed and supervised the manuscript. All authors contributed to the article and approved the submitted version.
