# Supplementary material for: The efficacy and safety of lebrikizumab monotherapy for the management of moderate-to-severe atopic dermatitis: A systematic review and meta-analysis
Source: Front Med (Lausanne). 2023 Jan 16;9:1091271. doi: 10.3389/fmed.2022.1091271 (PMC9884690; doi:10.3389/fmed.2022.1091271)
Supplement: Supplementary file 1 [file Data_Sheet_1.DOCX]

Atopic Dermatitis OR Eczema OR Atopic Allergies OR

Monoclonal Antibodies OR IL-13 inhibitors OR lebrikizumab

Randomized Controlled trial OR Randomized clinical trial OR Clinical trial OR Trial

CENTRAL = 129

Embase = 50

Medline = 92

CT.gov = 27

CENTRAL and Embase search strategy

Search History: 1/10/2022

ID Search

#1 MeSH descriptor: [Dermatitis, Atopic] explode all trees

#2 MeSH descriptor: [Eczema] explode all trees

#3 #1 OR #2

#4 MeSH descriptor: [Antibodies, Monoclonal] explode all trees

#5 (IL-13 inhibitors):ti,ab,kw (Word variations have been searched)

#6 (lebrikizumab):ti,ab,kw

#7 #4 OR #5 OR #6

#8 MeSH descriptor: [Randomized Controlled Trial] explode all trees

#9 MeSH descriptor: [Clinical Trial] explode all trees

#10 ("randomized controlled trial"):ti,ab,kw OR ("study"):ti,ab,kw OR (Trial):ti,ab,kw OR (RCT):ti,ab,kw (Word variations have been searched)

#11 #8 OR #9 OR #10

#12 #3 AND #7 AND #11

Medline Search Strategy

| Search | Query | Results |
| --- | --- | --- |
| #4 | Search: #1 AND #2 AND #3 | [92](https://pubmed.ncbi.nlm.nih.gov/?term=%231+AND+%232+AND+%233&sort=relevance&ac=no) |
| #3 | Search: **((((clinical trials, randomized[MeSH Terms]) OR (controlled clinical trials, randomized[MeSH Terms])) OR (randomized controlled trial[MeSH Terms])) OR (clinical trial[MeSH Terms]))** | [377,039](https://pubmed.ncbi.nlm.nih.gov/?term=%28%28%28%28clinical+trials%2C+randomized%5BMeSH+Terms%5D%29+OR+%28controlled+clinical+trials%2C+randomized%5BMeSH+Terms%5D%29%29+OR+%28randomized+controlled+trial%5BMeSH+Terms%5D%29%29+OR+%28clinical+trial%5BMeSH+Terms%5D%29%29&sort=date&ac=no) |
| #2 | Search: **((antibodies, monoclonal[MeSH Terms]) OR (lebrikizumab)) OR (IL-13 inhibitors)** | [271,136](https://pubmed.ncbi.nlm.nih.gov/?term=%28%28antibodies%2C+monoclonal%5BMeSH+Terms%5D%29+OR+%28lebrikizumab%29%29+OR+%28IL-13+inhibitors%29&sort=date&ac=no) |
| #1 | Search: **(((((atopic dermatitis[MeSH Terms]) OR (atopic dermatitides[MeSH Terms])) OR (allergic eczematous dermatitides[MeSH Terms])) OR (allergic eczematous dermatitis[MeSH Terms])) OR (atopic eczema[MeSH Terms])) OR (dermatitides, allergic eczematous[MeSH Terms])** | [36,278](https://pubmed.ncbi.nlm.nih.gov/?term=%28%28%28%28%28atopic+dermatitis%5BMeSH+Terms%5D%29+OR+%28atopic+dermatitides%5BMeSH+Terms%5D%29%29+OR+%28allergic+eczematous+dermatitides%5BMeSH+Terms%5D%29%29+OR+%28allergic+eczematous+dermatitis%5BMeSH+Terms%5D%29%29+OR+%28atopic+eczema%5BMeSH+Terms%5D%29%29+OR+%28dermatitides%2C+allergic+eczematous%5BMeSH+Terms%5D%29&sort=relevance&ac=no) |

CT.gov search strategy

Condition: Atopic Dermatitis OR Eczema OR Atopic Allergies OR Atopy

Other terms: Monoclonal Antibodies OR IL-13 inhibitors OR lebrikizumab

Search Limits: Study Type [interventional] Study results [with results]
